# Increased levels of sphingosine-1-phosphate in cerebrospinal fluid of patients diagnosed with tick-borne encephalitis

**DOI:** 10.1186/s12974-014-0193-4

**Published:** 2014-11-25

**Authors:** Alina Kułakowska, Fitzroy J Byfield, Małgorzata Żendzian-Piotrowska, Joanna M Zajkowska, Wiesław Drozdowski, Barbara Mroczko, Paul A Janmey, Robert Bucki

**Affiliations:** Department of Neurology, Medical University of Białystok, ul. M.Skłodowskiej-Curie 24a, Białystok, 15-276 Poland; Institute for Medicine and Engineering, University of Pennsylvania, 1010 Vagelos Research Laboratories, 3340 Smith Walk, Philadelphia, PA 19104 USA; Department of Physiology, Medical University of Bialystok, ul. Mickiewicza 2C, Białystok, 15-230 Poland; Department of Infectious Diseases and Neuroinfections, Medical University of Białystok, ul. Zurawia 14, Białystok, 15-345 Poland; Department of Neurodegeneration Diagnostics, Medical University of Białystok, ul. Waszyngtona 15, Białystok, 15-230 Poland; Department of Physiology, Pathophysiology and Microbiology of Infections, The Faculty of Health Sciences of the Jan Kochanowski University in Kielce, Kielce, Al. IX Wieków Kielc 19, Kielce, 25-317 Poland; Department of Microbiological and Nanobiomedical Engineering, Medical University of Bialystok, ul. Mickiewicza 2C, Białystok, 15-222 Poland

**Keywords:** Cerebrospinal fluid, Tick-borne encephalitis, Sphingosine-1-phosphate, IL-6, Gelsolin, FTY720P

## Abstract

**Background:**

Tick-borne encephalitis (TBE) is a serious acute central nervous system infection that can result in death or long-term neurological dysfunctions. We hypothesize that changes in sphingosine-1-phosphate (S1P) concentration occur during TBE development.

**Methods:**

S1P and interleukin-6 (IL-6) concentrations in blood plasma and cerebrospinal fluid (CSF) were measured using HPLC and ELISA, respectively. The effects of S1P on cytoskeletal structure and IL-6 production were assessed using rat astrocyte primary cultures with and without addition of plasma gelsolin and the S1P receptor antagonist fingolimod phosphate (FTY720P).

**Results:**

We report that acute inflammation due to TBE virus infection is associated with elevated levels of S1P and IL-6 in the CSF of infected patients. This elevated concentration is observed even at the earliest neurologic stage of disease, and may be controlled by glucocorticosteroid anti-inflammatory treatment, administered to patients unresponsive to antipyretic drugs and who suffer from a fever above 39°C. *In vitro*, treatment of confluent rat astrocyte monolayers with a high concentration of S1P (5 μM) results in cytoskeletal actin remodeling that can be prevented by the addition of recombinant plasma gelsolin, FTY720P, or their combination. Additionally, gelsolin and FTY720P significantly decreased S1P-induced release of IL-6.

**Conclusions:**

TBE is associated with increased concentration of S1P and IL-6 in CSF, and this increase might promote development of inflammation. The consequences of increased extracellular S1P can be modulated by gelsolin and FTY720P. Therefore, blocking the inflammatory response at sites of infection by agents modulating S1P pathways might aid in developing new strategies for TBE treatment.

## Background

Sphingosine-1-phosphate (S1P) is a product of sphingomyelin (SM) metabolism. It is present in most eukaryotic organisms. S1P regulates cell function both intracellularly and by binding to extracellular receptors [1]. As an extracellular mediator, S1P binds to a family of G-protein-coupled receptors named S1P(1)-S1P(5) and has multiple physiological effects [2]. In the immune system, cell surface S1P(1) receptors transduce the rapid, transient effects of extracellular S1P on T- and B-lymphocyte trafficking, promote early T-cell migration to tissue sites of immune responses, and regulate T-cell proliferation and secretion of numerous cytokines [1,3]. S1P receptors are also found on all cell types in the CNS, and the effects of S1P on neurons [4], astrocytes [5,6], oligodendrocytes [7], and microglia [8,9] are highly cell-type specific. For example, S1P is a chemoattractant for neural precursor cells and is proposed to direct migration of neurons to sites of injury [10]. Additionally, S1P [7] or synthetic ligands of S1P receptors [11,12] interact with neurotrophin-3 to promote survival of oligodendrocytes.

S1P is abundant in plasma where it is bound to high-density lipoproteins and albumin [13]. Recently we have shown that plasma gelsolin, an actin-binding PIP_2_-regulated protein, can also act as a universal carrier or scavenger of S1P, and its function may include interference with S1P actions [14]. Such an interaction may be of importance in settings where the concentrations of both substances change over their homeostatic ranges. Multiple sclerosis (MS), a chronic immune-mediated inflammatory disease of the CNS, seems to be one example of such a condition [15]. In the CSF of patients with MS, S1P and gelsolin concentrations showed a tendency to increase and decrease, respectively, when compared to other non-inflammatory neurological disorders [15]. An inflammatory reaction accompanied by central nervous system (CNS) infections such as tick-borne encephalitis (TBE) or Lyme neuroborreliosis (LNB), also results in blood and cerebrospinal fluid (CSF) alterations in plasma gelsolin [15]. TBE, a systemic infection with RNA virus leads to the development of an acute meningitis and encephalitis, characterized by swelling of the brain due to inflammation [16]. Even though TBE can be prevented by active immunization, it is still very common in some regions of the world, such as Central Europe, and currently no specific treatment is known [17,18]. We hypothesize that TBE may result in an alteration of S1P concentration in the blood and CSF of patients, and in such a case modulation of S1P cellular effects may be used to develop new treatment strategies. FTY720P, a S1P receptor modulator, was found to be highly effective in the treatment of MS [19], and its immunomodulatory activity may be potentially beneficial in other CNS inflammatory conditions.

## Materials and methods

### Materials

S1P (S9666) was purchased from Sigma Aldrich (St Louis, Missouri, United States). (S)- fingolimod phosphate (FTY720P; B-0721) was purchased from Echelon Biosciences Inc. (Salt Lake City, Utah, United States). Recombinant human plasma gelsolin (rhGSN) was obtained from Biogen-Idec Inc (Cambridge, Massachusetts, United States). Stock solutions of S1P were prepared in 0.3 M NaOH, since pH strongly affects the aggregation behavior of S1P [20]. Various concentrations of S1P were prepared by mixing its sonicated stock solution; 10 minutes, at room temperature (RT) with buffer required for a particular experiment.

### Specimen collections

Human blood and CSF specimen collection was performed in the Department of Neurology and Department of Infectious Diseases and Neuroinfections at the Medical University of Białystok (Poland). Blood was collected in heparinized syringes and centrifuged at 1,500 g for 5 minutes at 4°C. The protocol for this study was approved by the Ethics Committee for Research on Humans, Medical University of Białystok (approval number: R-I-002/382/2012). At the time of patient recruitment, written consent was obtained from all subjects. All individuals were undergoing lumbar puncture for diagnostic purposes, and most of the TBE patients received a second lumbar puncture 10 to 12 days later to monitor the course of the disease. Following lumbar punctures, CSF cells were counted, and the rest of the material (after centrifugation: 2,000 g for 10 minutes), along with plasma samples, were frozen and kept at −80°C.

Clinical and laboratory characteristics of the patient groups are given in Table 1. Briefly, all TBE patients were divided into the following groups: without and with glucocorticosteroids (GCs) treatment (+G), from whom the CSF and plasma were collected at admission (TBE-I/TBE + G-I; patients at time of admission and first lumbar puncture without or with GCs treatment respectively) and patients from whom the CSF and plasma samples were collected after 10 to 12 days of treatment (TBE-II/TBE + G-II; patients after 10 to 12 days of hospitalization, non-treated or treated with GCs, respectively). Diagnosis of TBE was confirmed by detection of anti-TBE virus antibodies in the serum and CSF by ELISA (Virion-SERION kit, SERION ELISA classic TBE Virus IgG/IgM,Würzburg, Germany). All TBE patients were given symptomatic treatment (analgesics, antipyretics, and intravenous rehydration). Additionally, patients in poor general condition (fever above 39°C and seizures or disturbances of consciousness (Glasgow Coma Scale score <12 points)) were treated with glucocorticosteroid (dexamethasone 12 to 16 mg daily), which was given intravenously during a period of four to six days.

**Table 1:** **Clinical and laboratory characteristics of the patient groups**

**Clinical group**	**Total number**	**Age**		**CSF**	
	**(Females)**	**(Years)**	**QALB**	**Total protein (μg/ml)**	**Lymphocytes**
**TBE-I**					
with GCs treatment	21(10)	43.7 ± 16.2	12.1 ± 1.4	851 ± 121	105 ± 15.1
without GCs treatment	24(11)			829 ± 129	93 ± 12.1
**TBE-II**		40.7 ± 16.9	11.3 ± 1.7		
with GCs treatment	19(10)			811 ± 128	76 ± 23.4
without GCs treatment		44.2 ± 12.8	11.6 ± 1.3	784 ± 122	76 ± 14.1
	20(9)				
**LNB**		39.1 ± 16.7	10.8 ± 1.3	782 ± 152	48.2 ± 9.3
	18(7)	46.5 ± 10.3	9.8 ± 1.1		
**MS**	22(16)	37.2 ± 13.4	6.5 ± 0.9	435 ± 163	5.7 ± 4.6
**Control**					
Idiopathic cephalgia	21(14)	43.6 ± 21.4	6.1 ± 0.5	414 ± 144	3.7 ± 1.9
Idiopathic (Bell’s) facial nerve palsy	16(10)	45.2 ± 18.8	6.7 ± 1.1	375 ± 183	4.1 ± 1.2

QALB = albumin concentration in CSF: albumin concentration in blood. CSF – cerebrospinal fluid, TBE-I – tick borne encephalitis before treatment, TBE-II – tick borne encephalitis after 10 to 12 days of treatment, LNB – Lyme neuroborreliosis, MS – multiple sclerosis, GCs – glucocorticosteroids.

LNB was diagnosed according to the European Federation of Neurological Societies criteria [21]. In all our LNB patients the diagnosis was ‘definite neuroborreliosis’ and all of them suffered from meningitis, which is a typical clinical manifestation of early LNB. The ELISA method (Borrelia recombinant IgG/IgM ELISA, Vienna, Austria) and immunoblotting (LINE Virotech, Rüsselsheim, Germany) were used to detect antibodies against *Borrelia burgdorferi* in serum and CSF of LNB patients. MS patients included in the study were in the process of MS diagnosis, and their EDSS (Expanded Disability Status Scale) scores were between 0.5 and 4.0 (1.7 ± 0.9). Finally, they were diagnosed according to McDonald’s criteria [22] as relapsing-remitting MS. At the time of lumbar puncture none of the MS patients was treated with steroids or any disease-modifying drugs. Control patients without infection were diagnosed with conditions such as idiopathic cephalgia and idiopathic Bell’s facial nerve palsy, in which standard CSF diagnostic tests show no abnormalities.

### Evaluation of sphingosine-1-phosphate in blood plasma and cerebrospinal fluid samples

S1P concentration was measured by the method described in Min *et al*. [23]. Briefly, acidified methanol and internal standard (30 pmol of C_17_-S1P, Avanti Polar Lipids, Inc., Alabaster, USA) were added to 250 μl of plasma or CSF, and the samples were ultrasonicated in ice-cold water for one minute. Lipids were then extracted by the addition of chloroform, 1 M NaCl, and 3 N NaOH. The alkaline aqueous phase containing S1P was transferred to a fresh tube. The residual S1P in the chloroform phase was re-extracted twice with methanol/1 M NaCl (1:1, v/v) solution, and then all the aqueous fractions were combined. The amount of S1P was determined indirectly after dephosphorylation to sphingosine, with the use of alkaline phosphatase (bovine intestinal mucosa, Fluka, Milwaukee, Washington, United States). To improve the extraction yield of released sphingosine, chloroform was carefully placed at the bottom of the reaction tubes. The chloroform fraction containing the dephosphorylated sphingoid base was washed three times with alkaline water (pH adjusted to 10.0 with ammonium hydroxide) and then evaporated under a nitrogen stream. The dried lipid residues were re-dissolved in ethanol, converted to their o-phthalaldehyde derivatives, and analyzed using an HPLC system (ProStar, Varian Inc. Walnut Creek, USA) equipped with a fluorescence detector and a C18 reversed-phase column (OmniSpher 5, 4.6150 mm, Varian Inc. Walnut Creek, USA). The isocratic eluent composition of acetonitrile/water (9:1, v/v) and a flow rate of 1 ml/min were used. Column temperature was maintained at 33°C by use of a column oven (Varian Inc. Walnut Creek, USA).

### Cell culture study

Mixed cortical cultures were obtained from prenatal rats and maintained for 14 days, or until confluence was reached, in Neurobasal™ media supplemented with 0.4 M GlutaMax and B27 (50× dilution) (Life Technologies, 3175 Staley Road, Grand Island, NY 14072, USA) at 37°C and 5% CO_2_. Neuronal cells were removed through a series of trypsinizations, and the remaining astrocytes were maintained at 37°C and 5% CO2 in DMEM + GlutaMax (Life Technologies, 3175 Staley Road, Grand Island, NY 14072, USA) supplemented with 5% fetal bovine serum. The purity of astrocyte cultures was 80% or more, as determined by immunofluorescence staining for glial fibrillary acidic protein [24,25]. In all experiments, the medium was changed to serum-free 6 to 12 hours prior to S1P, GSN, or FTY720P addition. Rat astrocytes were treated for 8 hours, fixed with 4% paraformaldehyde, permeabilized with 0.1% Triton X-100 and stained for F-actin with Phalloidin-FITC (Life Technologies, 3175 Staley Road, Grand Island, NY 14072, USA). Microscopic evaluation was performed using a Leica microscope (Leica Microsystems Inc., 1700 Leider Lane Buffalo Grove, IL 60089, USA) (40× objective) and images captured with a Hamamatsu camera (Hamamatsu, Eastern Regional Office, 250 Wood Avenue Middlesex, NJ 08846, USA). To evaluate GSN and FTY720P effects on interleukin-6 (IL-6) release from rat astrocytes, cells were activated for 8 hours. Cell-free supernatants were collected by centrifugation at 5,000 × g for 5 minutes and stored at −80°C until cytokine determination. IL-6 was measured using a sandwich enzyme-linked immunosorbent assay (ELISA Kits, DRG Interleukin-6 (human) Marburg, Germany), according to the manufacturer’s instructions.

### Statistical analysis

Data are reported as a mean ± SE (standard error) or SD (standard deviation). Data analysis was performed using one-way analysis of variance (ANOVA) tests with a *post-hoc* Bonferroni analysis test. Differences between means were evaluated using the Student’s t-test, with *P* <0.05 being taken as the level of significance.

## Results and discussion

**Figure 1:**
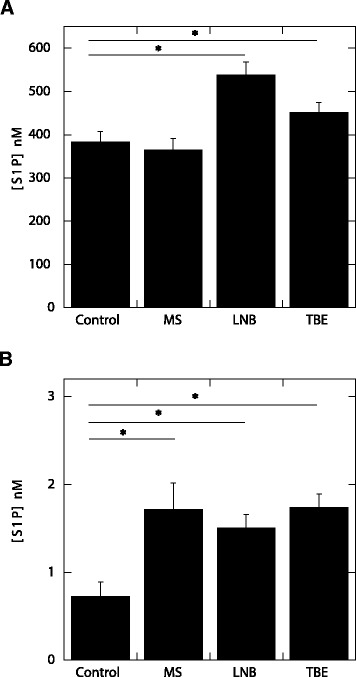
**S1P concentration in blood plasma (A) and CSF (B) samples obtained from control (n =37) subjects and from patients with various inflammatory diseases of the CNS: MS (n =22), LNB (n =18), TBE (n =45).** *Significantly different. Error bars represent standard error of the mean. S1P – sphingosine – 1– phosphate, CSF – cerebrospinal fluid, MS – multiple sclerosis, LNB – Lyme neuroborreliosis, TBE – tick borne encephalitis.

**Figure 2:**
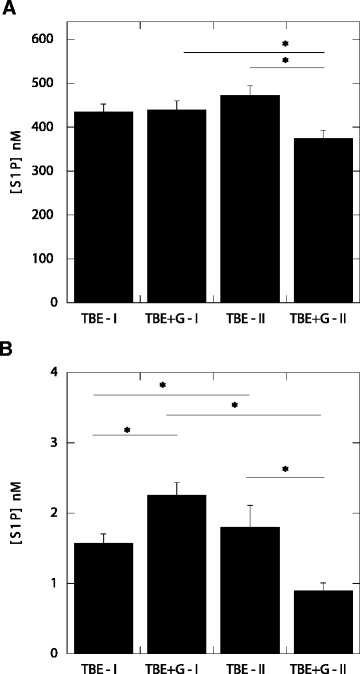
**S1P concentration in blood plasma (A) and CSF (B) samples obtained from TBE patients.** TBE-I (n =24) and TBE + G-I (n =21) samples obtained before treatment from subjects not treated and treated with glucocorticosteroids, respectively. TBE-II (n =20) and TBE + G-II (n =19) samples obtained 10 to 12 days later from patients not treated and treated with glucocorticosteroids, respectively. *Significantly different. Error bars represent standard error of the mean. S1P – sphingosine – 1– phosphate, CSF – cerebrospinal fluid, TBE – tick borne encephalitis.

Using HPLC methods, we found that the average S1P concentration in plasma of TBE (451.0 ± 21.8 nM) and LNB (538.0 ± 30.5 nM) patients was significantly higher (*P* <0.001) than in control subjects (383 ± 23.3 nM) (Figure 1). There were no differences between average S1P concentration in the plasma of control and MS patients. Average S1P concentrations in the CSF of patients with inflammatory diseases of the CNS (MS 1.71 ± 0.29 nM, LNB 1.5 ± 0.14 nM, TBE 1.74 ± 0.14 nM) were also significantly higher (*P* <0.001) compared to the control group (0.72 ± 0.16 nM). These results are in close agreement with our previous study in which we showed an intrathecal increase of S1P in early stage MS [26]. They also confirm our preliminary finding of an increase of S1P concentration in lymphocytic meningitis [14]. Comparing S1P concentration at different stages of TBE, as shown in Figure 2, we found that a short course of glucocorticosteroid treatment resulted in a significant decrease of S1P concentration in the blood (*P* <0.02) and CSF (*P* <0.05) of TBE patients. More precisely, before treatment S1P levels were 433.2 ± 19.2 nM and 2.29 ± 0.22 nM in plasma and CSF, respectively, and after treatment they were 370 ± 24.9 nM and 0.95 ± 0.21 nM in plasma and CSF, respectively. Average S1P concentration in plasma of TBE patients not treated with glucocorticosteroids remained stable (439.4 ± 21.0 nM versus 446 ± 35.8 nM). However, in the CSF of TBE subjects not treated with glucocorticosteroids, S1P concentration continued to increase during the course of the disease and was significantly higher (*P* <0.05) in samples obtained during the second lumbar puncture performed 10 to 12 days after the first, compared to the samples taken during the first lumbar puncture, which was performed at the time of diagnosis (1.79 ± 0.39 nM versus 1.48 ± 0.23 nM)*.* This difference suggests an essential role of S1P in progression of these inflammatory processes. The initial S1P concentration in the CSF of subjects in poor general condition treated with glucocorticosteroids was higher compared to the initial S1P concentration in non-treated patients who were in better general condition. We did not observe significant changes in basic CSF parameters after glucocorticosteroid treatment (Table 1). However, the decrease in pleocytosis, which is an indicator of inflammation, was greater in treated subjects. The mean difference between the first and second lumbar puncture (number of lymphocytes in 1 μl of CSF) was 29 and 17 in treated and non-treated patients, respectively.

There is currently no specific treatment for TBE infection, and treatment with glucocorticosteroids is still used in the most severe cases in countries with a high prevalence of the disease [27,28]. In the north-eastern part of Poland (region of Bialystok) the incidence of the disease is 5.1 to 13.1 out of 100,000, and patients from this region constitute more than 40% of all TBE cases reported in the whole country [25]. A retrospective study conducted at the Departments of Infectious Diseases and Neuroinfections, Medical University of Białystok (Poland) included 687 patients diagnosed with TBE and hospitalized in this department between 1993 and 2008. About 60% (407 of 687) of them were given dexamethasone, with clinical improvement in most cases, and no serious side effects were observed [25]. However, treatment with glucocorticosteroids is controversial [29,30] and may lead to serious metabolic and circulatory complications. Therefore, this therapy should be reserved only for certain severe cases of the disease, in which probability of clinical benefit is higher than risk of serious adverse events. There is a need for new effective and safe medications.

**Figure 3:**
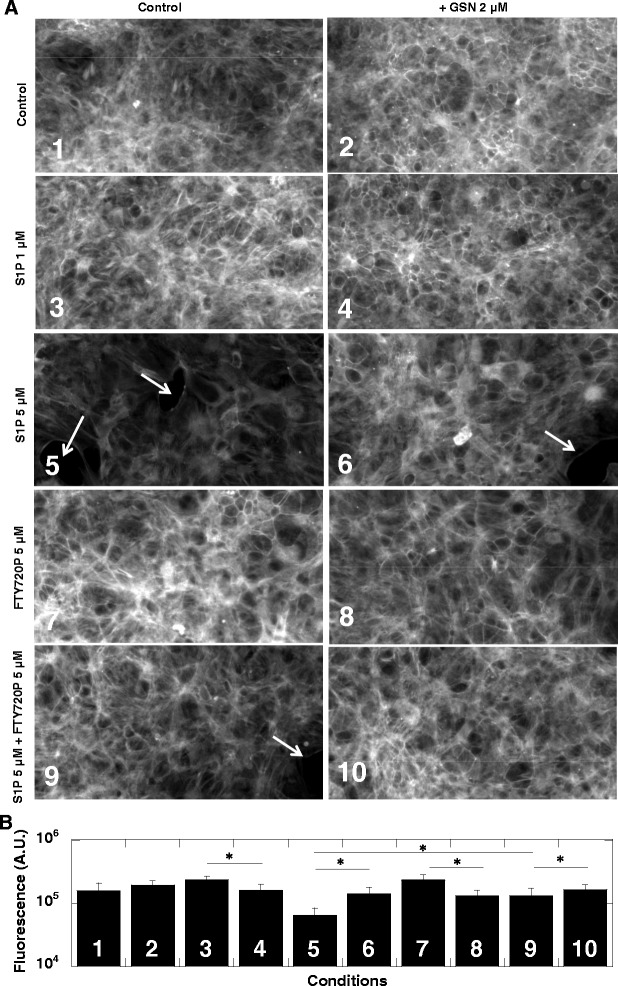
**F-actin structure in rat astrocytes (confluent culture) treated with S1P, recombinant human plasma gelsolin and FTY720P, or their combination.** At 8 hours incubation the cells were fixed with 4% paraformaldehyde, permeabilized with Triton X-100 and F-actin was stained with Phalloidin-FITC. Arrows indicate gaps in astrocyte monolayers. Data from one representative experiment are shown **(A)**. **B** shows quantitative analysis of F-actin fluorescence in the astrocyte monolayer under treatment, indicated by numbers 1 to 10 in the left lower corner of each picture. *Significantly different. Error bars represent standard error of the mean. S1P – sphingosine – 1– phosphate, GSN – recombinant human plasma gelsolin.

Astrocytes are the most abundant cells in the brain [31] and changes in their F-actin cytoskeleton organization occur during pro-inflammatory cytokine secretion [32], as well as changes in the regulatory interface at the blood–brain barrier [33]. Accordingly, following inflammatory stimuli, significant changes in astrocyte actin filament organization have been observed [14,31]. Results from our cell culture studies indicate that factors interfering with S1P signaling affect astrocyte F-actin cytoskeletal organization. As shown in Figure 3A, a low concentration of S1P (1 μM) and recombinant human gelsolin (2 μM), added separately, did not have a dramatic effect on F-actin organization, but 1 μM S1P in combination with GSN produced a marked increase in the F-actin staining at the cytoplasmic margin (cortical F-actin), associated with a significant decrease of total F-actin (Figure 3B). In contrast, a high concentration (5 μM) of S1P produced a decrease in total F-actin concentration. Additionally, gaps comparable to the size of an astrocyte were observed in the monolayer after this treatment. These results may explain the severe blood-CSF barrier disruption in our TBE patients in whom the QALB (QALB = albumin concentration in CSF: albumin concentration in blood) ratio, an indicator of blood-CSF barrier function, was definitely above norm (norm for 40-year-old individuals is about 6.5) [34]. At admission, patients treated and not treated with glucocorticosteroids had similar QALB ratios; 12.1 ± 1.4 and 11.3 ± 1.7, respectively. Indeed, there is evidence to suggest that weakening of the blood–brain barrier may precede, accelerate, or contribute to a number of neurodegenerative disorders, and that a gap formation may lead to changes in the blood-CSF barrier function [35,36]. On the other hand, in the cell culture system, the effect of high S1P treatment was inhibited by co-treatment with gelsolin, 5 μM FTY720P, or a combination of gelsolin and FTY720P. Interestingly, treatment with 5 μM FTY720P alone caused a significant increase in F-actin, which was slightly inhibited by gelsolin.

**Figure 4:**
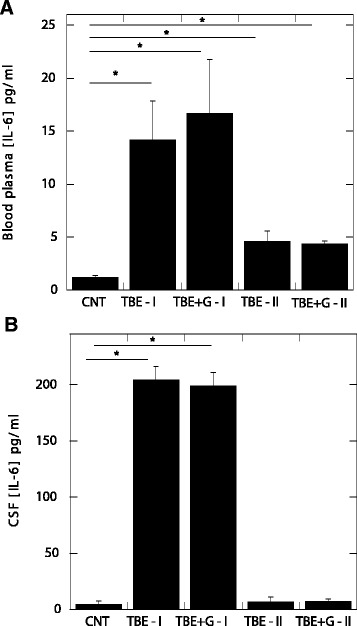
**IL-6 concentration in blood plasma (panel A) and cerebrospinal fluid (panel B) obtained from control (CNT, n =6) and TBE patients.** TBE-I (n =9) and TBE + G-I (n =10) samples obtained before treatment from subjects not treated and treated with glucocorticosteroids, respectively. TBE-II (n =9) and TBE + G-II (n =10) samples obtained 10 to 12 days later from patients not treated and treated with glucocorticosteroids, respectively. *Significantly different. Error bars represent standard deviation of the mean. IL–6 – interleukin 6, TBE – tick borne encephalitis.

Astrocytes release a variety of cytokines in response to CNS infections and injuries [37,38]. IL-6 is a pleiotropic pro-inflammatory cytokine produced by astrocytes that may also act as a trophic factor in the nervous system [39-41]. Mechanisms that regulate IL-6 expression require activation of p38 mitogen-activated protein kinases and depend on NF-κB transcriptional activity. Initiation of these pathways in astrocytes occurs when the PI3K-mTOR-AKT pathway is inhibited [42]. As indicated by data shown in Figure 4, a statistically significant increase of IL-6 in blood and CSF was observed in samples collected from TBE subjects at the time of diagnosis, compared to controls (Figure 4). This observation confirmed our previous study [43]. Moreover, in an animal model of TBE, significantly increased IL-6 mRNA was found, but only in mice susceptible to the infection. In the resistant strain, upregulation of IL-6 mRNA expression was not observed. These data suggest that excessive production of pro-inflammatory mediators, such as IL-6, may contribute to development of a more severe form of TBE [44].

**Figure 5:**
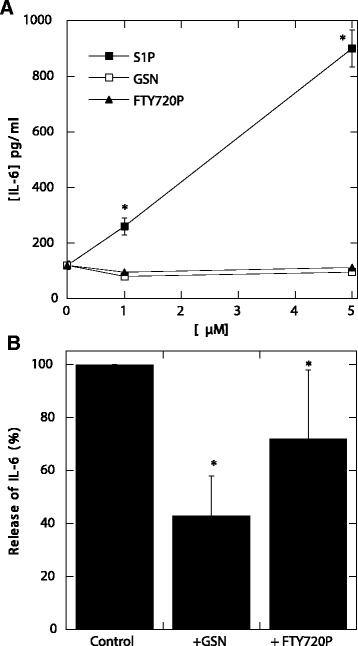
**S1P, recombinant human plasma gelsolin (rhGSN) and FTY720P prevent release of IL-6 from rat astrocytes.** IL-6 release from rat astrocytes 8 hours after addition of S1P, (rhGSN and FTY720P **(A)**. Decrease of IL-6 release from rat astrocytes activated with S1P (5 μM) in the presence of rhGSN (5 μM) and FTY720P (5 μM) **(B)**. *Significantly different. Error bars represent standard deviation of the mean. IL–6 – interleukin 6, S1P – shingosine-1-phosphate

Our results indicate that S1P, but not GSN or FTY720-P, induced production of IL-6, and treatment with GSN or FTY720-P inhibited IL-6 release by rat astrocytes stimulated with S1P (Figure 5). These results suggest that the S1P pathway may partly govern the production of pro-inflammatory cytokines by astrocytes. Consequently, it is highly probable that the potential therapeutic effects of GSN (which has a significantly decreased concentration in the blood of TBE patients [45]) and FTY720P might be caused by modulation of S1P-mediated activation of astrocytes in the CNS. It was recently reported that FTY720 exposure might regulate specific neuroinflammatory responses by desensitizing astrocytes to external S1P [19]. Studies in experimental autoimmune encephalomyelitis using mice with conditionally deleted S1P(1) receptor from astrocytes indicate that one beneficial effect of FTY720P in this model occurs via downregulation of external receptors, which inhibits responses induced by the natural agonist. Another proposed effect of FTY720P on neuroinflammation is its ability to maintain persistent signaling in cells via internalized S1P(1) receptor resulting in functional responses that include suppressing intracellular calcium release [19]. Further *in vivo* experiments with FTY720P would be required to demonstrate a causal role of S1P in the pathogenesis of TBE.

## Conclusions

Acute CNS inflammation due to TBE virus infection is associated with an elevated S1P concentration in the blood and CSF of infected patients. In a cell culture system, the functions of astrocytes in an inflammatory state, induced by a high concentration of S1P, can be restored to their normal state by the administration of rhGSN, FTY720P, or their combination. This observation could be of clinical significance and might be useful for developing new treatments for pathological CNS conditions associated with rising concentrations of S1P in the blood and CSF, such as are produced by TBE.

## Abbreviations

CSF: Cerebrospinal fluid
FTY720P: Fingolimod phosphate
GCs: Glucocorticosteroids
GSN: Gelsolin
IL-6: Interleukin-6
LNB: Lyme neuroborreliosis
MS: Multiple sclerosis
S1P: Sphingosine-1-phosphate
TBE: Tick-borne encephalitis

## References

[1] Goetzl EJ, Wang W, McGiffert C, Liao JJ, Huang MC (2007). Sphingosine 1-phosphate as an intracellular messenger and extracellular mediator in immunity. Acta Paediatr Suppl.

[2] Hla T (2003). Signaling and biological actions of sphingosine 1-phosphate. Pharmacol Res.

[3] Huang MC, Graeler M, Shankar G, Spencer J, Goetzl EJ (2002). Lysophospholipid mediators of immunity and neoplasia. Biochim Biophys Acta.

[4] Konno N, Nakamura A, Ikeno Y, Cheon SH, Kitamoto K, Arioka M (2007). Novel neurotrophic effects of sphingosylphosphorylcholine in cerebellar granule neurons and in PC12 cells. Biochem Biophys Res Commun.

[5] Mullershausen F, Craveiro LM, Shin Y, Cortes-Cros M, Bassilana F, Osinde M, Wishart WL, Guerini D, Thallmair M, Schwab ME, Sivasankaran R, Seuwen K, Dev KK (2007). Phosphorylated FTY720 promotes astrocyte migration through sphingosine-1-phosphate receptors. J Neurochem.

[6] Osinde M, Mullershausen F, Dev KK (2007). Phosphorylated FTY720 stimulates ERK phosphorylation in astrocytes via S1P receptors. Neuropharmacology.

[7] Saini HS, Coelho RP, Goparaju SK, Jolly PS, Maceyka M, Spiegel S, Sato-Bigbee C (2005). Novel role of sphingosine kinase 1 as a mediator of neurotrophin-3 action in oligodendrocyte progenitors. J Neurochem.

[8] Tham CS, Lin FF, Rao TS, Yu N, Webb M (2003). Microglial activation state and lysophospholipid acid receptor expression. Int J Dev Neurosci.

[9] Schilling T, Repp H, Richter H, Koschinski A, Heinemann U, Dreyer F, Eder C (2002). Lysophospholipids induce membrane hyperpolarization in microglia by activation of IKCa1 Ca(2+)-dependent K(+) channels. Neuroscience.

[10] Kimura A, Ohmori T, Ohkawa R, Madoiwa S, Mimuro J, Murakami T, Kobayashi E, Hoshino Y, Yatomi Y, Sakata Y (2007). Essential roles of sphingosine 1-phosphate/S1P1 receptor axis in the migration of neural stem cells toward a site of spinal cord injury. Stem Cells.

[11] Coelho RP, Payne SG, Bittman R, Spiegel S, Sato-Bigbee C (2007). The immunomodulator FTY720 has a direct cytoprotective effect in oligodendrocyte progenitors. J Pharmacol Exp Ther.

[12] Miron VE, Jung CG, Kim HJ, Kennedy TE, Soliven B, Antel JP (2008). FTY720 modulates human oligodendrocyte progenitor process extension and survival. Ann Neurol.

[13] Bartke N, Hannun YA (2009). Bioactive sphingolipids: metabolism and function. J Lipid Res.

[14] Bucki R, Kulakowska A, Byfield FJ, Zendzian-Piotrowska M, Baranowski M, Marzec M, Winer JP, Ciccarelli NJ, Gorski J, Drozdowski W, Bittman R, Janmey PA (2010). Plasma gelsolin modulates cellular response to sphingosine 1-phosphate. Am J Physiol Cell Physiol.

[15] Kulakowska A, Ciccarelli NJ, Wen Q, Mroczko B, Drozdowski W, Szmitkowski M, Janmey PA, Bucki R (2010). Hypogelsolinemia, a disorder of the extracellular actin scavenger system, in patients with multiple sclerosis. BMC Neurol.

[16] Zambito Marsala S, Pistacchi M, Gioulis M, Mel R, Marchini C, Francavilla E (2014). Neurological complications of tick borne encephalitis: the experience of 89 patients studied and literature review. Neurol Sci.

[17] Schosser R, Reichert A, Mansmann U, Unger B, Heininger U, Kaiser R (2014). Irregular tick-borne encephalitis vaccination schedules: The effect of a single catch-up vaccination with FSME-IMMUN. A prospective non-interventional study. Vaccine.

[18] Heinz FX, Stiasny K, Holzmann H, Grgic-Vitek M, Kriz B, Essl A, Kundi M (2013). Vaccination and tick-borne encephalitis, central Europe. Emerg Infect Dis.

[19] Wu C, Leong SY, Moore CS, Cui QL, Gris P, Bernier LP, Johnson TA, Seguela P, Kennedy TE, Bar-Or A, Antel JP (2013). Dual effects of daily FTY720 on human astrocytes in vitro: relevance for neuroinflammation. J Neuroinflammation.

[20] Sasaki H, Arai H, Cocco MJ, White SH (2009). pH dependence of sphingosine aggregation. Biophys J.

[21] Mygland A, Ljostad U, Fingerle V, Rupprecht T, Schmutzhard E, Steiner I (2010). EFNS guidelines on the diagnosis and management of European Lyme neuroborreliosis. Eur J Neurol.

[22] Polman CH, Reingold SC, Banwell B, Clanet M, Cohen JA, Filippi M, Fujihara K, Havrdova E, Hutchinson M, Kappos L, Lublin FD, Montalban X, O'Connor P, Sandberg-Wollheim M, Thompson AJ, Waubant E, Weinshenker B, Wolinsky JS (2011). Diagnostic criteria for multiple sclerosis: 2010 revisions to the McDonald criteria. Ann Neurol.

[23] Min JK, Yoo HS, Lee EY, Lee WJ, Lee YM (2002). Simultaneous quantitative analysis of sphingoid base 1-phosphates in biological samples by o-phthalaldehyde precolumn derivatization after dephosphorylation with alkaline phosphatase. Anal Biochem.

[24] Drogemuller K, Helmuth U, Brunn A, Sakowicz-Burkiewicz M, Gutmann DH, Mueller W, Deckert M, Schluter D (2008). Astrocyte gp130 expression is critical for the control of Toxoplasma encephalitis. J Immunol.

[25] Antanitus DS, Choi BH, Lapham LW (1975). Immunofluorescence staining of astrocytes in vitro using antiserum to glial fibrillary acidic protein. Brain Res.

[26] Kulakowska A, Zendzian-Piotrowska M, Baranowski M, Kononczuk T, Drozdowski W, Gorski J, Bucki R (2010). Intrathecal increase of sphingosine 1-phosphate at early stage multiple sclerosis. Neurosci Lett.

[27] Czupryna P, Moniuszko A, Pancewicz SA, Grygorczuk S, Kondrusik M, Zajkowska J (2011). Tick-borne encephalitis in Poland in years 1993–2008–epidemiology and clinical presentation. A retrospective study of 687 patients. Eur J Neurol.

[28] Mickiene A, Laiskonis A, Gunther G, Vene S, Lundkvist A, Lindquist L (2002). Tickborne encephalitis in an area of high endemicity in lithuania: disease severity and long-term prognosis. Clin Infect Dis.

[29] Schut ES, Brouwer MC, de Gans J, Florquin S, Troost D, van de Beek D (2009). Delayed cerebral thrombosis after initial good recovery from pneumococcal meningitis. Neurology.

[30] Spapen H, Van Berlaer G, Moens M, Hubloue I (2011). Adjunctive steroid treatment in acute bacterial meningitis. “To do or not to do: that is the question”. Acta Clin Belg.

[31] Min KJ, Yang MS, Kim SU, Jou I, Joe EH (2006). Astrocytes induce hemeoxygenase-1 expression in microglia: a feasible mechanism for preventing excessive brain inflammation. J Neurosci.

[32] Block L, Bjorklund U, Westerlund A, Jorneberg P, Biber B, Hansson E (2013). A new concept affecting restoration of inflammation-reactive astrocytes. Neuroscience.

[33] Pottiez G, Sevin E, Cecchelli R, Karamanos Y, Flahaut C (2009). Actin, gelsolin and filamin-A are dynamic actors in the cytoskeleton remodelling contributing to the blood brain barrier phenotype. Proteomics.

[34] Reiber H, Peter JB (2001). Cerebrospinal fluid analysis: disease-related data patterns and evaluation programs. J Neurol Sci.

[35] Rist RJ, Romero IA, Chan MW, Couraud PO, Roux F, Abbott NJ (1997). F-actin cytoskeleton and sucrose permeability of immortalised rat brain microvascular endothelial cell monolayers: effects of cyclic AMP and astrocytic factors. Brain Res.

[36] Deli MA, Descamps L, Dehouck MP, Cecchelli R, Joo F, Abraham CS, Torpier G (1995). Exposure of tumor necrosis factor-alpha to luminal membrane of bovine brain capillary endothelial cells cocultured with astrocytes induces a delayed increase of permeability and cytoplasmic stress fiber formation of actin. J Neurosci Res.

[37] Lieberman AP, Pitha PM, Shin HS, Shin ML (1989). Production of tumor necrosis factor and other cytokines by astrocytes stimulated with lipopolysaccharide or a neurotropic virus. Proc Natl Acad Sci U S A.

[38] Gottschall PE, Tatsuno I, Arimura A (1994). Regulation of interleukin-6 (IL-6) secretion in primary cultured rat astrocytes: synergism of interleukin-1 (IL-1) and pituitary adenylate cyclase activating polypeptide (PACAP). Brain Res.

[39] Kahn MA, De Vellis J (1994). Regulation of an oligodendrocyte progenitor cell line by the interleukin-6 family of cytokines. Glia.

[40] Guptarak J, Wanchoo S, Durham-Lee J, Wu Y, Zivadinovic D, Paulucci-Holthauzen A, Nesic O (2013). Inhibition of IL-6 signaling: a novel therapeutic approach to treating spinal cord injury pain. Pain.

[41] Cui X, Liu J, Bai L, Tian J, Zhu J (2014). Interleukin-6 induces malignant transformation of rat mesenchymal stem cells in association with enhanced signaling of signal transducer and activator of transcription 3. Cancer Sci.

[42] Codeluppi S, Fernandez-Zafra T, Sandor K, Kjell J, Liu Q, Abrams M, Olson L, Gray NS, Svensson CI, Uhlen P (2014). Interleukin-6 secretion by astrocytes is dynamically regulated by PI3K-mTOR-calcium signaling. PLoS One.

[43] Zajkowska J, Grygorczuk S, Pryszmont JM, Kondrusik M, Pancewicz S, Swierzbinska R, Hermanowska-Szpakowicz T, Klibingat M (2006). Concentration of interleukin 6 and 10 in tick-borne and purulent encephalomeningitis. Pol Merkur Lekarski.

[44] Palus M, Vojtiskova J, Salat J, Kopecky J, Grubhoffer L, Lipoldova M, Demant P, Ruzek D (2013). Mice with different susceptibility to tick-borne encephalitis virus infection show selective neutralizing antibody response and inflammatory reaction in the central nervous system. J Neuroinflammation.

[45] Kulakowska A, Zajkowska JM, Ciccarelli NJ, Mroczko B, Drozdowski W, Bucki R (2011). Depletion of plasma gelsolin in patients with tick-borne encephalitis and Lyme neuroborreliosis. Neurodegener Dis.

